# Tyrosine 51 residue of the syndecan-2 extracellular domain is involved in the interaction with and activation of pro-matrix metalloproteinase-7

**DOI:** 10.1038/s41598-019-47140-5

**Published:** 2019-07-23

**Authors:** Bohee Jang, Ji-Hye Yun, Sojoong Choi, Jimin Park, Dong Hae Shin, Seung-Taek Lee, Weontae Lee, Eok-Soo Oh

**Affiliations:** 1 From the Department of Life Sciences, the Research Center for Cellular Homeostasis, Ewha Womans University, Seoul, 120-750 Republic of Korea; 2 Department of Biochemistry, College of Life Science and Biotechnology, Yonsei University, Seoul, 120-749 Republic of Korea; 3 College of Pharmacy, Ewha Womans University, Seoul, 120-750 Republic of Korea

**Keywords:** Solution-state NMR, Extracellular matrix

## Abstract

Although syndecan-2 is known to interact with the matrix metalloproteinase-7 (MMP-7), the details of their interaction were unknown. Our experiments with a series of syndecan-2 extracellular domain deletion mutants show that the interaction is mediated through an interaction of the extracellular domain of syndecan-2 (residues 41 to 60) with the α2 helix-loop-α3 helix in the pro-domain of MMP-7. NMR and molecular docking model show that Glu7 of the α1 helix, Glu32 of the α2 helix, and Gly48 and Ser52 of the α2 helix-loop-α3 helix of the MMP-7 pro-domain form the syndecan-2-binding pocket, which is occupied by the side chain of tyrosine residue 51 (Tyr51) of syndecan-2. Consistent with this notion, the expression of a syndecan-2 mutant in which Tyr51 was changed to Ala diminished the interaction between the syndecan-2 extracellular domain and the pro-domain of MMP-7. Furthermore, HT-29 colon adenocarcinoma cells expressing the interaction-defective mutant exhibited reductions in the cell-surface localization of MMP-7, the processing of pro-MMP-7 into active MMP-7, the MMP-7-mediated extracellular domain shedding of both syndecan-2 and E-cadherin, and syndecan-2-mediated anchorage-independent growth. Collectively, these data strongly suggest that Tyr51 of the syndecan-2 extracellular domain mediates its interaction with and activating processing of pro-MMP-7 and regulates MMP-7-dependent syndecan-2 functions.

## Introduction

The extracellular matrix (ECM) is the three-dimensional network that structurally and functionally integrates cells into tissues^1^. Through its diversity in composition and physical nature, the ECM can perform many functions, such as providing support, separate tissues, and regulating intercellular communication. Therefore, the dysregulation of ECM homeostasis is closely associated with pathological conditions and can exacerbate the progression of many different diseases, including pulmonary fibrosis, systemic sclerosis, liver cirrhosis, and cardiovascular disease^2^.

During cancer development, the ECM is commonly altered via changes in the synthesis or degradation of one or more ECM components. This affects the progression of cancer by directly promoting cell transformation and altering the composition of the ECM; thus, ECM remodeling is crucial for tumorigenesis and metastatic progression^2–4^.

The matrix metalloproteinases (MMPs) perform important protease functions in ECM degradation and remodeling^5,6^. Of them, matrix metalloproteinase-7 (MMP-7) has been well studied for its roles in cancer progression. MMP-7 is overexpressed in a variety of epithelial cancers, such as stomach^7^, liver^8^, pancreatic^9^, and colon^10^ cancer. As seen for other MMPs, increased MMP-7 regulates cancer progression and invasion through regulating the proteolytic degradation of ECM molecules (e.g., elastin, type IV collagen, fibronectin, vitronectin, aggrecan, and proteoglycan)^11^, and non-ECM molecules (e.g., β4 integrin, E-cadherin, FasL, proHB-EGF, and TNFα precursor)^12^. Due to these varied impacts, high-level MMP-7 expression is associated with poor survival^13^. Since all MMPs are synthesized and secreted as pro-enzymes, the activation of pro-MMP-7 is a critical step in the degradation of MMP-7 substrates. The cell-surface localization of MMP-7 may be a key event in providing its proteolytic activity with the ability to promote ECM degradation and tumor invasion. Indeed, studies have shown that the localization of MMPs on the cell surface is of great importance for their carcinogenesis-related processing and activity regulation. For example, MMP-2 can be localized to the cell surface through interactions with integrin αvβ3^14^ or MT1-MMP^15^, while heparan sulfate proteoglycan, CD44, is responsible for docking MMP-7 and −9^13^ to the cell surface. In human colon cancer cells, our group previously showed that syndecan-2 (SDC-2), another cell surface heparan sulfate proteoglycan (HSPG), interacts with MMP-7 to ensure its localization to the cell surface^16^. Syndecan-2 may interact with MMP-7 through either/both glycosaminoglycan chains^17,18^ or/and core proteins^16^. However, the mechanism by which pro-MMP-7 is activated and localized to the cell surface remains unknown.

In general, the syndecans mediate the interaction between the ECM and the cell, and thus physically link the ECM, cytoskeleton, and assembly of the adhesion-signaling complex^19^. The members of the syndecan family are cell-surface adhesion receptors whose extracellular domains interact with many kinds of extracellular ligands to transduce signals from the extracellular environment to the cytosol^20^. The syndecans regulate these interactions, and thereby contribute to regulating various processes, including development, wound healing, and differentiation of neural and glioma stem cells^21,22^. Our group previously reported that syndecan-2 regulates MMP-7 gene expression in colon cancer cells^16^, indicating that syndecan-2 in association with MMP-7 regulates colon cancer activities. Syndecans can also regulate extracellular signals as docking receptors by interacting with extracellular ligands, such as growth factors, cytokines, chemokines, and MMPs^23,24^. For example, syndecans help FGF bind to FGF receptor with higher affinity^23^. Serglycin and versican core protein have been shown to interact with the pro-form of MMP-9, and these interactions were found to modulate the activation and substrate binding of pro-MMP-9^25,26^. In addition, the MMP-9/HSPG complex is known to be concentrated at the highly metastatic cell leading edge, where it is critical for the metastasis of murine colon adenocarcinoma cells^27^. We previously showed that syndecan-2 directly interacts with pro-MMP-7 and may contribute to processing it into active MMP-7 in colon cancer cells^16^. Therefore, the function(s) of MMP and cell surface receptors appear to be closely correlated.

Given that syndecan-2 and MMP-7 are important regulators in colon carcinogenesis^13,28,29^ and we previously showed that the extracellular domain of syndecan-2 interacts with pro-MMP-7 to cooperatively regulate tumorigenic activities in human colon cancer cells, we herein further investigated the detailed structural basis of the interaction between the syndecan-2 extracellular domain and the pro-domain of MMP-7. Our results suggest that syndecan-2-mediated regulation of cancer activity depends on its interaction with and activation of MMP-7.

## Results

### The N-terminal syndecan-2 extracellular domain directly interacts with the MMP-7 pro-domain

**Figure 1:**
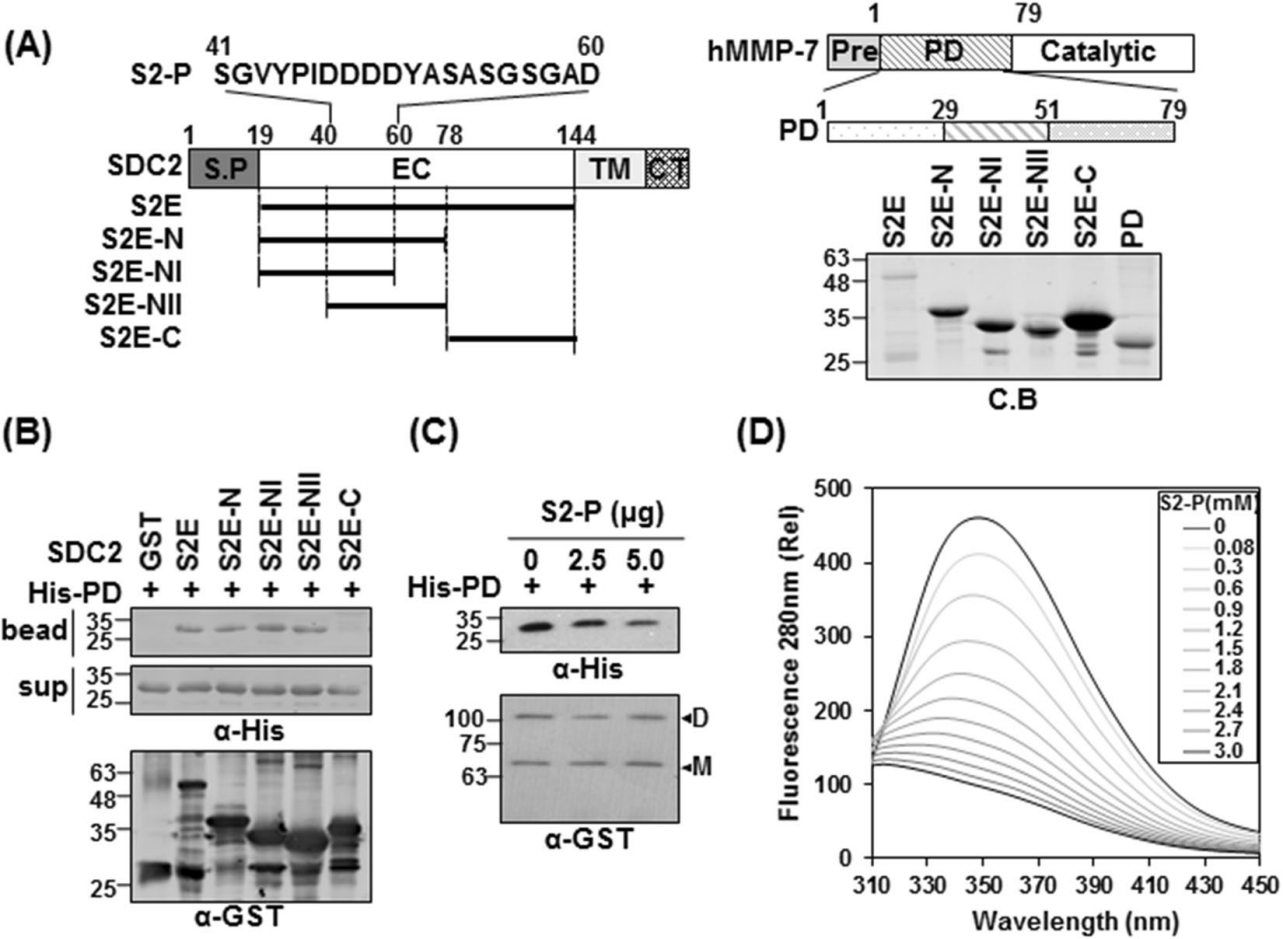
The N-terminus of syndecan-2 interacts with the pro-domain of MMP-7. (**A**) Schematic representation of the syndecan-2 core protein (SDC2) and MMP-7. The signal peptide (SP), the extracellular domain (EC), the transmembrane domain (TM), and the cytoplasmic domain (CT) of syndecan-2 are noted, and the various deletion mutants are indicated. A peptide corresponding to residues 41–60 of the syndecan-2 extracellular domain (S2-P) was synthesized. Syndecan-2 is labeled with amino acid numbers to show the location of each deletion (left). Schematic representation of MMP-7. The pre-domain (Pre), pro-domain (PD), and catalytic domain are shown (right top). Purified GST-SDC2 mutants and the His-tagged pro-domain of MMP-7 were separated by 15% SDS-PAGE and stained with Coomassie Blue (right bottom). (**B**) Purified GST or GST-SDC2 mutants were incubated with His-tagged pro-domain of MMP-7 (His-PD). Bound materials were subjected to immunoblotting with an anti-His tag antibody (top). The membranes were then stripped and re-probed with an anti-GST antibody (bottom). (**C**) Purified GST-SDC2 was incubated with purified His-PD MMP-7 plus the indicated amounts of S2-P for 2 h at 4 °C. Bound materials were subjected to immunoblotting with an anti-His tag antibody (top). The membranes were then stripped and re-probed with an anti-GST antibody (bottom). M and D indicate monomer and dimer of syndecan-2, respectively. (**D**) Fluorescence spectroscopy indicates the binding affinity between His-PD and S2-P peptide. Titration between His-PD and S2-P peptide was performed up to 1 by 200 molar ratios and the K_*d*_ value was calculated as 1.586 ± 0.012 mM.

We previously reported that rat syndecan-2 N-terminal extracellular domain interacts with MMP-7^16^. To further explore the interaction of human syndecan-2 and MMP-7, we generated additional deletion mutants of the GST-tagged extracellular domain of human syndecan-2 and the His-tagged pro-domain of MMP-7 (PD). We then purified recombinant GST-syndecan-2 core protein mutants using glutathione agarose beads (Fig. 1A) and performed *in vitro* binding assays with purified His-tagged pro-domain of MMP-7 (His-PD). Our results revealed that GST-tagged syndecan-2 extracellular domain (S2E) interacted with the pro-domain of MMP-7, as did the GST-tagged N-terminal extracellular domain of syndecan-2 (S2E-N, amino acid residues 19–78), but not the C-terminal extracellular domain of syndecan-2 (S2E-C) (Fig. 1B). This suggests that the interaction site resides in the N-terminus of the extracellular domain. Interestingly, both of the tested N-terminal deletion mutants (S2E-NI and -NII) interacted with His-PD (Fig. 1B), further suggesting that amino acid residues 41–60 of the human syndecan-2 extracellular domain are involved in the interaction with pro-domain of MMP-7. Consistent with these findings, a synthetic human syndecan-2 peptide (S2-P) dose-dependently inhibited the interaction of GST-syndecan-2 and His-PD (Fig. 1C). Fluorescence tryptophan quenching assays showed that S2-P peptide dose-dependently interacted with His-PD with a Kd value of 1.586 ± 0.012 mM (Fig. 1D). These data suggest that amino acids 41–60 in the N-terminal region of the human syndecan-2 extracellular domain are responsible for the interaction of syndecan-2 with the pro-domain of MMP-7.

### Helix 2-helix 3 of the pro-domain of MMP-7 contributes to the interaction with the N-terminus of the syndecan-2 extracellular domain

**Figure 2:**
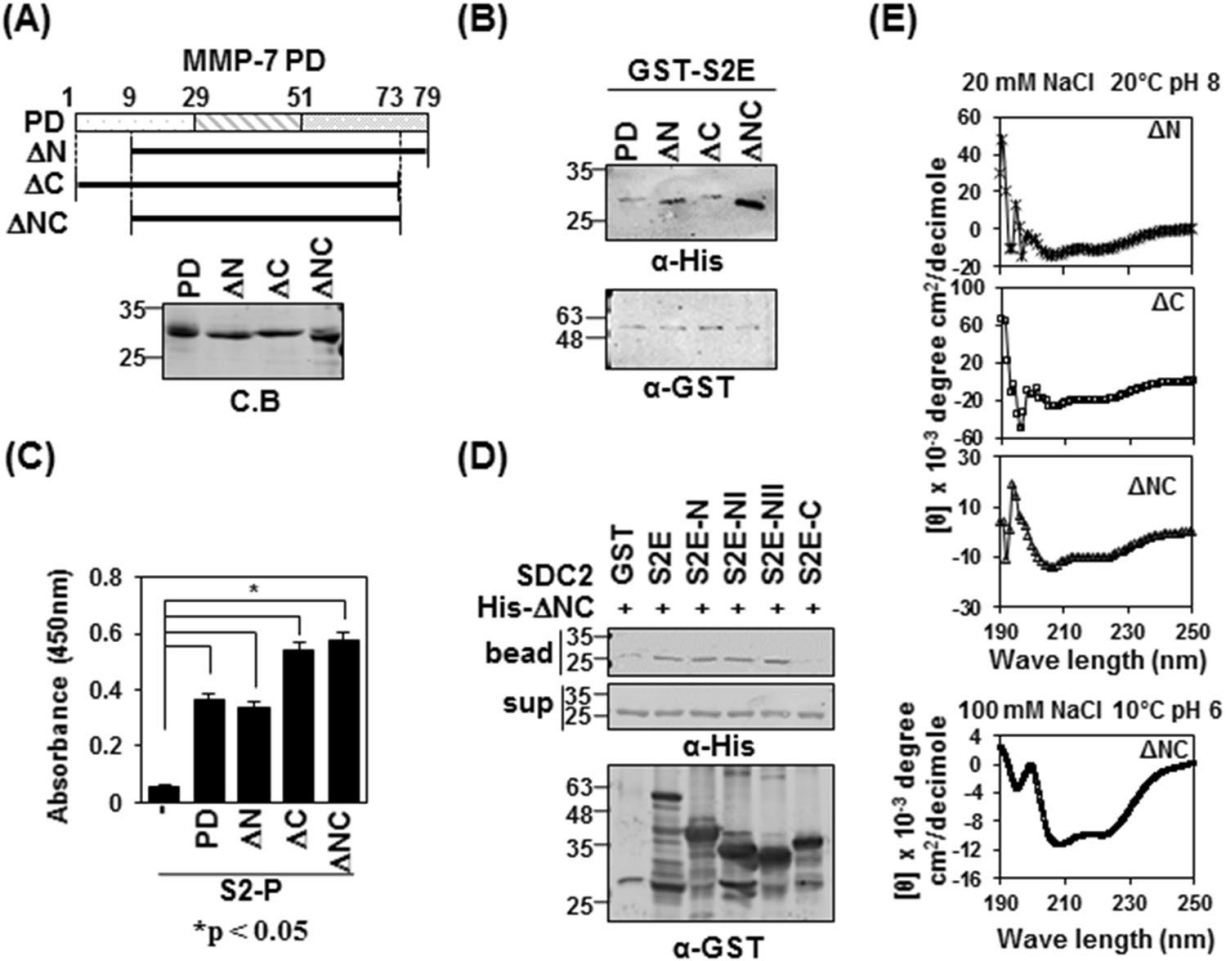
The pro-domain of MMP-7 is involved in its interaction with the syndecan-2 extracellular domain. (**A**) Schematic representations of the pro-domain of MMP-7 (PD) and the deletion mutants lacking the pro-domain, N-terminus (ΔN), C-terminus (ΔC) and both N- and C-terminus (ΔNC) (top). His-tagged MMP-7 pro-domain was purified with Ni-NTA agarose beads, separated by SDS-PAGE and stained with Coomassie Blue (bottom). (**B**) Purified GST-S2E, S2E-C and S2E-NII were incubated with His-tagged PD, or ΔN, ΔC or ΔNC MMP-7 pro-domain. Materials bound to glutathione-agarose beads were immunoblotted with an anti-His tag antibody (top). The membranes were then stripped and re-probed with an anti-GST antibody (bottom). (**C**) ELISA plates coated with 600 ng of S2-P were incubated with the indicated His-tagged MMP-7 pro-domains (500 ng/well) for 2 h at 20 °C. The plates were washed, incubated with an anti-His tag antibody followed by IgG-HRP, and developed with TMB-ELISA. Absorbance was measured at 450 nm. Data are shown as mean ± S.D. (n = 3), *p < 0.05 versus blank. (**D**) Purified GST-SDC2 was mixed with His-tagged ΔNC for 2 h at 4 °C., separated by SDS-PAGE, and immunoblotted with anti-His or -GST antibodies. (**E**) The secondary structures of the MMP-7 pro-domain mutants of ΔN, ΔC and ΔNC were diluted to 50 μM in a buffer consisting of 10 mM HEPES and 20 mM NaCl, pH 8, and kept at 20 °C. The TFE-induced helicity curves were obtained by recording the CD signal for each independent sample from 0% to 40% TFE (100 mM NaCl, pH 6, 20 °C) with far-UV.

The pro-domain of MMP-7 is composed of three α-helical domains connected by flexible linkers (SWISS-MODEL and sequence alignment). To map the syndecan-2-interacting site in the pro-domain of MMP-7, we generated MMP-7 pro-domain mutants lacking the N-terminal linker of the pro-domain (ΔN, residues 9–79), the C-terminal linker (ΔC, residues 1–73), or both (ΔNC, residues 9–73, Fig. 2A). Our *in vitro* pulldown assay showed that all three deletion mutants interacted with GST-tagged extracellular domain of syndecan-2 (S2E) (Fig. 2B), confirming that there is a direct interaction between syndecan-2 and the pro-domain of MMP-7. Interestingly, the S2E protein interacted more strongly with mutants lacking both linkers (ΔNC) (Fig. 2B). Consistently, when pro-domain mutants were incubated on syndecan-2 peptide (S2-P)-coated ELISA plates, all of the pro-domain mutants showed interactions with S2-P (Fig. 2C). All syndecan-2 mutants containing the amino acid sequence of the S2-P peptide interacted with ΔNC (Fig. 2D), suggesting that mutants with deletion of the C-terminal linker might have a more stable conformation. Indeed, our analysis of circular dichroism (CD) spectra showed that whereas ΔN and ΔC presented with mixtures of random coils and helical structures, ΔNC had the most stable helical structure among the pro-domain mutants, as evidenced by the appearance of double minima, with negative peaks seen at 208 and 222 nm (Fig. 2E).

**Figure 3:**
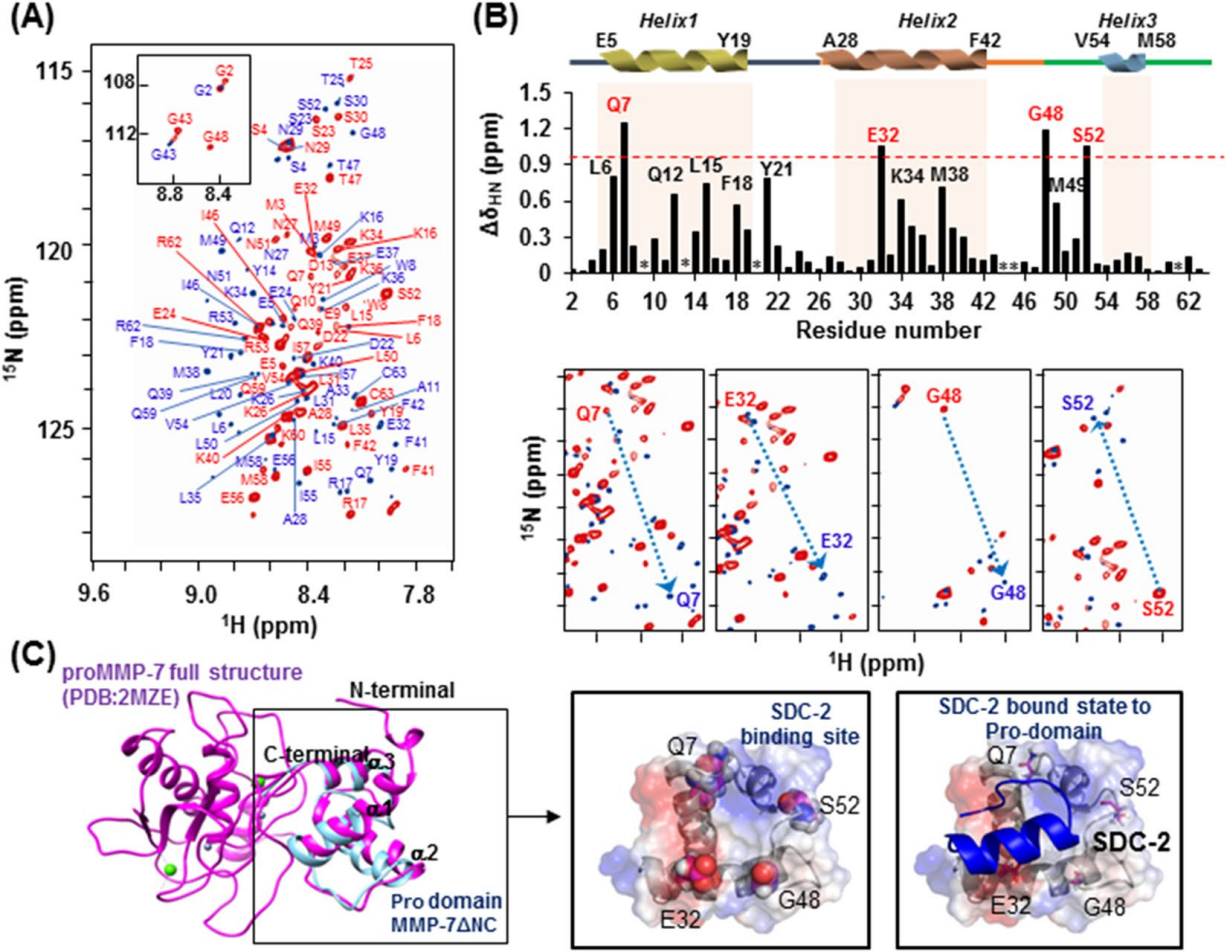
Chemical shift perturbations of the ^1^HN and ^15^N resonances of proMMP-7 ΔNC upon binding the human syndecan-2 peptide ligand. (**A**) ^1^H-^15^N HSQC spectrum of proMMP-7 ΔNC in the absence and presence of S2-P. The spectra were collected at pH 6 using Bruker 850 and 900 MHz spectrometers, and NMR titrations were performed with ^15^N-labeled proMMP-7 ΔNC and S2-P at a molar ratio of 1:15. The backbone resonance assignments are shown in red (1:0) and blue (1:15). (**B**) The chemical shift perturbations of proMMP-7 ΔNC in the absence and presence of S2-P were assessed by overlapping the three titration points and performing interaction-site mapping of the proMMP-7/S2-P peptide complex using NMR titration (top). The average chemical-shift changes were calculated using the following formula: Δδ_AV_ = [(Δδ_1H_)^2^ + (Δδ_15N_/5)^2^]^1/2^, where Δδ_AV_, Δδ_1H_, and Δδ_15N_ represent the average chemical shift value, proton chemical shifts, and nitrogen chemical shift changes, respectively. The highly perturbed residues, Gln 7, Glu 32, Gly 48, and Ser 52, are marked in red. The chemical-shift movements of these residues were plotted in overlapped HSQC analyses (bottom). (**C**) The NMR structure of the pro-domain of human MMP-7 (PDB; 2MZE) (shown in magenta) and the modeled structure of proMMP-7 ΔNC (shown in cyan) were overlapped and visualized using a ribbon diagram (left). The side-chains of the four highly perturbed residues (Gln 7, Glu 32, Gly 48, and Ser 52) are shown as balls (center). HADDOCK model obtained for the proMMP-7/S2-P complex based on our NMR titration data, including the predicted binding site for S2-P on the pro-domain of proMMP-7 (right).

To further analyze the interaction between the extracellular domain of syndecan-2 and the pro-domain of MMP-7, we performed nuclear magnetic resonance (NMR) experiments, including ^1^H-^15^N heteronuclear single quantum coherence (HSQC), HNCACB, and CBCACONH, using single- and double-labeled ΔNC (Fig. 3). The residual backbone in the absence (marked by red) and presence (marked by blue) of S2-P was interpreted by following the correlation of the NH group with the preceding Cα and Cβ chemical shifts. We found that titration of S2-P triggered large chemical shift changes in the trend of the 2D ^1^H-^15^N HSQC spectrum. On the 2D ^1^H-^15^N HSQC, the peaks gathered near 8.4–8.6 ppm were broadened by the addition of S2-P, which means that the pro-domain of MMP-7 became well folded upon its combination with S2-P (Fig. 3A). When we used overlapping 2D-HSQC to analyze the chemical-shift perturbation upon peptide binding, we found that four residues were highly perturbed during NMR titration experiments performed with S2-P: Gln7, Glu32, Gly48 and Ser52 (Fig. 3B, upper). With the exception of Gly48, these residues are more likely to exhibit an electrostatic charge-based interaction through their side chains when combining with S2-P. Eight additional residues were somewhat perturbed during these experiments: Leu6, Gln12, Leu15 and Phe18 located in the α1 helix; Tyr21 located in α1 helix-loop-α2 helix; Lys34 and Met38 located in the α2 helix; and Met49 located in the α2 helix-loop-α3 helix. However, the chemical shift perturbation of these residues seems to be involved in the hydrophobic interaction that stabilizes the structure while the pro-domain of MMP-7 binds to S2-P, rather than participating in the association with S2-P (Fig. 3B).

We calculated a predicted structural model for ΔNC using the TALOS program and secondary structure homology modeling analysis. Our results indicated that ΔNC retained the triple α-helical structure (Fig. 3C, left) found in the NMR structure previously published for the MMP-7 zymogen (PDB: 2MZE, 2MZH, 2MZI)^30^. Interestingly, the four residues found to be highly perturbed during NMR titration were all predicted to fit together on one surface of the pro-domain of MMP-7 (Fig. 3C; center). To predict the complex formed between the pro-domain of MMP-7 and syndecan-2, we performed simulation modeling using HEX 6.3^31^ and HADDOCK^32^. These analyses suggested that the S2-P interacting pocket also placed on the surface of the pro-domain of MMP-7, predicted by NMR titration (Fig. 3C, right).

**Figure 4:**
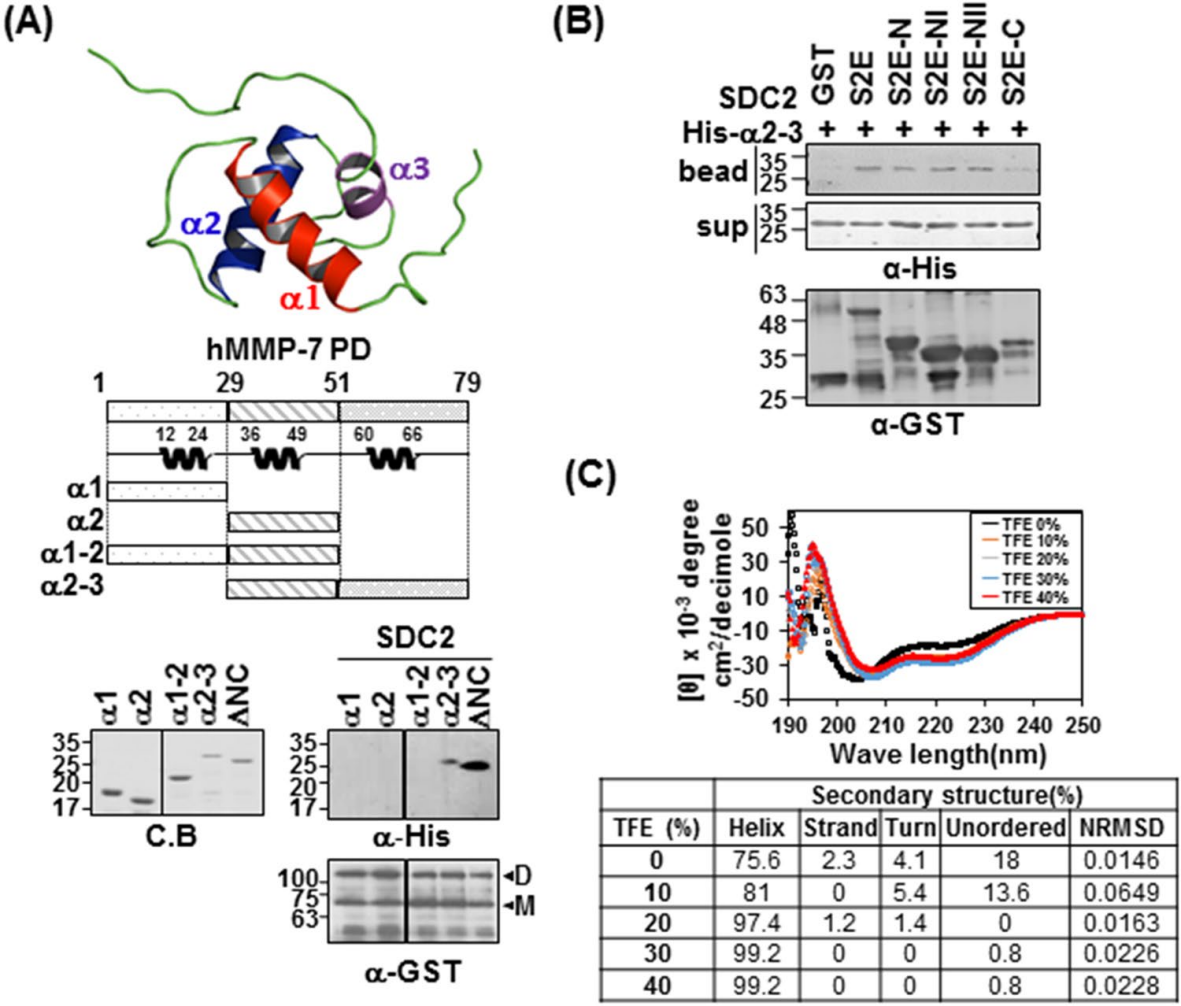
The α2–3 helix of the MMP-7 pro-domain is involved in its interaction with the syndecan-2 extracellular domain. (**A**) Structure of the MMP-7 pro-domain (PDB; 2MZE) and schematic diagram of the MMP-7 pro-domain (PD) and its deletion mutants. The indicated MMP-7 pro-domain mutants were purified with Ni-NTA agarose beads, separated by SDS-PAGE. Purified MMP-7 pro-domain mutants were incubated with GST-SDC-2. Materials bound to glutathione-agarose beads were immunoblotted with an anti-His tag antibody and the membranes were stripped and re-probed with an anti-GST antibody. Image is of a single membrane cropped to remove intervening lanes. (**B**) Purified GST-SDC2 mutants were incubated with His-tagged α2–3 helix of the MMP-7 pro-domain. Materials bound to glutathione-agarose beads were immunoblotted with an anti-His tag antibody (top) and the membranes were then stripped and re-probed with an anti-GST antibody (bottom). (**C**) The secondary structure of the α2–3 helix of the MMP-7 pro-domain was analyzed using CD in the presence of different concentrations of TFE (top). Quantitative estimations of the secondary-structure content were made with the CDPro software package, which includes the programs CDSSTR, CONTIN, and SELCON3. The α-helical fractions were extracted from the CDPro calculations based on empirical methods with ellipticities set at 208 or 222 nm (bottom). Smaller values of NRMSD indicate closer correspondence between calculated structures and the experimental data.

Since our NMR titration data suggest that the α1 helix, α2 helix, and α2 helix-loop-α3 helix may be involved in the interaction with syndecan-2, we further performed *in vitro* binding assays with each helical region of the pro-domain of MMP-7 (Fig. 4). As shown in Fig. 4A, the α2 helix-loop-α3 helix protein fragment (α2–3) interacted with syndecan-2, although to a slightly weaker degree than ΔNC; meanwhile, the other deletion mutants (α1, α2, α1–2) failed to interact. Consistent with this finding, α2–3 interacted with the N-terminus of the extracellular domain of syndecan-2 (Fig. 4B). These data suggest that the pro-domain MMP-7 α2–3 helix is involved in the interaction with the N-terminus of the syndecan-2 extracellular domain. CD spectra showed that, similar to ΔNC, α2–3 had a helical structure, as suggested by the presence of minima (negative peaks) at 208 and 222 nm; however, it appeared to have a lower degree of helicity (75%) (Fig. 4C). The addition of 2,2,2-trifluoroethanol (TFE), a well-known secondary structure-stabilizing agent^33^, dose-dependently stabilized the helicity of α2–3 by 75.6% to 99.2% depending on the TFE concentration (Fig. 4C), confirming that α2–3 formed a stable helical structure. Together, these data suggest that helix 2-helix 3 of the pro-domain of MMP-7 are involved in its interaction with the N-terminal extracellular domain of syndecan-2.

### Tyr51 in the syndecan-2 extracellular domain mediates the interaction with the pro-domain of MMP-7

**Figure 5:**
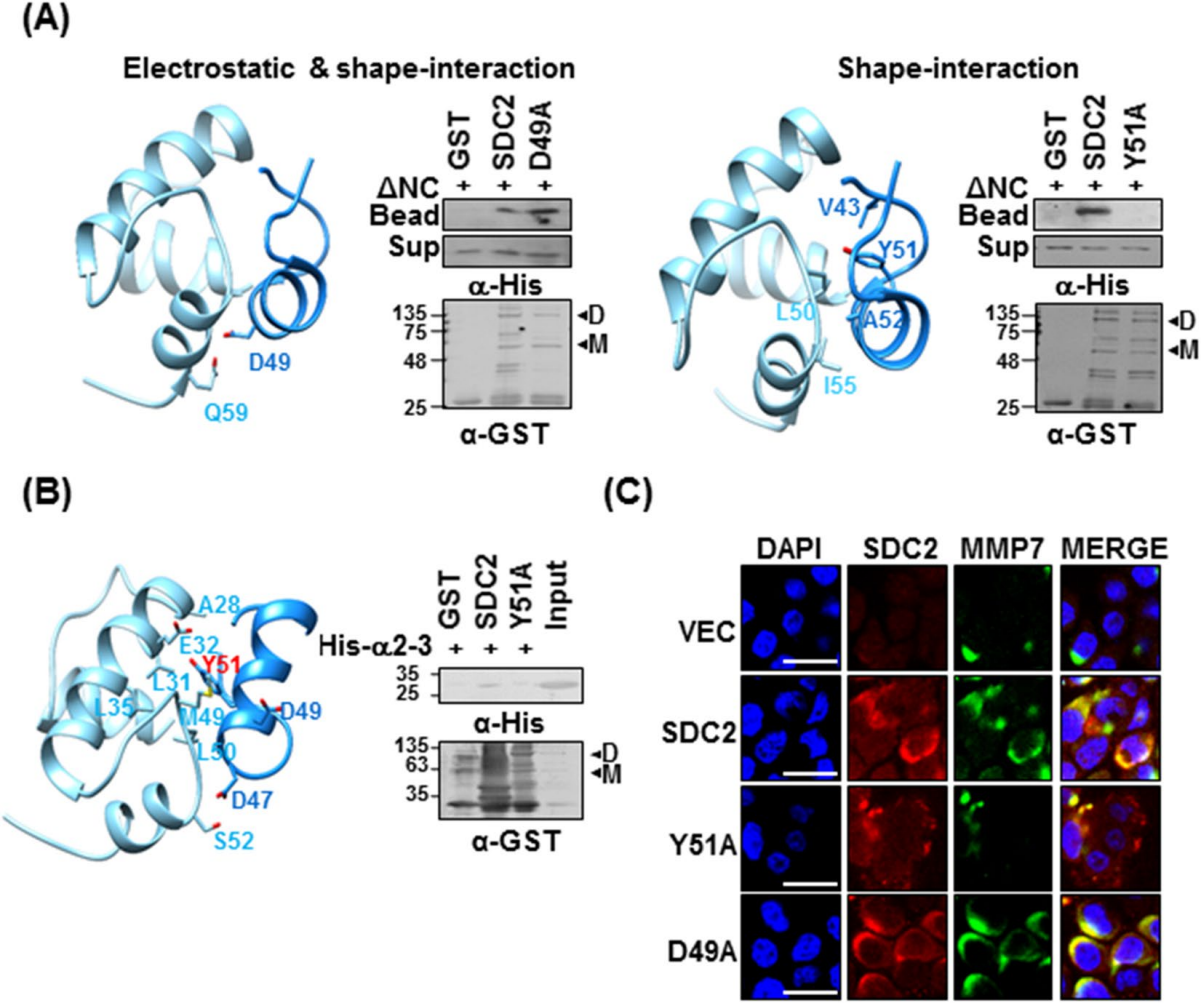
Tyrosine 51 of the syndecan-2 extracellular domain is involved in the interaction of syndecan-2 with the pro-domain of MMP-7. (**A**) A docking structure for the complex formed between ΔNC (sky blue) and S2-P (dodger blue), as generated by the HEX 6.3 program. Docking states were generated using electrostatic and shape interactions (a), or with shape interactions alone (b). Purified GST-SDC2 or its mutants were incubated with His-ΔNC for 2 h at 4 °C. Materials bound to glutathione-agarose beads were immunoblotted with the indicated antibodies (a or b right). (**B**) The HADDOCK program was used to generate the docking structure of ΔNC and S2-P. The residues identified as being important matched those identified in our NMR titration experiments. The residues lining the hydrophobic cavity are drawn with a stick model (left). Purified GST-SDC2 and the Y51A mutant were incubated with His-ΔNC for 2 h at 4 °C and the materials that bound to the glutathione-agarose beads were immunoblotted with the indicated antibodies (right). (**C**) Control HT-29 cells (VEC) and HT-29 cells transfected with vectors encoding SDC2 or mutants were immunostained with anti-syndecan-2 or anti-MMP-7. The results were visualized with Texas Red-conjugated goat anti-rabbit (red) or FITC-conjugated goat anti-mouse (green). DAPI was used to stain nuclei (blue). Scale bar, 20 μm.

To further investigate the detailed interaction mechanism of the pro-domain of MMP-7 and S2-P, we generated models of ΔNC and the syndecan-2 S2-P peptide and simulated their docking with the HEX 6.3^31^. The two candidate docking states were anticipated based on their electrostatic interaction and shape-interaction scores. Electrostatic plus shape-interaction scoring suggested that the S2-P peptide interacted with the α1–2 helix of pro-domain of MMP-7 via Asp49 of S2-P; however the substitution of Asp49 with Ala in the syndecan-2 extracellular domain (D49A) did not affect its interaction with His-tagged ΔNC (Fig. 5A, a). If we considered only the shape-interaction score, the α2–3 helix of pro-domain of MMP-7 was suggested to contribute to its interaction with S2-P peptide via Tyr51 of S2-P (Fig. 5A, a). The latter model structure suggests that there is hydrophobic pocket located between helices α2 and α3 of pro-domain of MMP-7, and that it receives the side chain of Tyr51 within the syndecan-2 extracellular domain (Fig. 5A, b). Consistent with this hypothesis and the involvement of Tyr51 in this interaction, the substitution of Tyr51 with Ala (Y51A) reduced the interaction of the syndecan-2 extracellular domain with His-tagged ΔNC (Fig. 5A, b). To further refine the docking model and perhaps explain the experimental NMR titration results, we used the HADDOCK program (Fig. 5B). The obtained model also suggests that Tyr51 and Asp47 of S2-P interact with Glu32 and Ser52 of pro-domain of MMP-7, respectively (Fig. 5B left). Moreover, while recombinant syndecan-2 interacted with the α2–3 of the pro-domain of MMP-7, the Y51A mutant failed to interact with the α2–3 (Fig. 5B, right), again suggesting that Tyr51 contributes to the interaction of syndecan-2 with the pro-domain of MMP-7.

We next investigated whether Tyr51 of syndecan-2 is involved in the cell-surface localization of MMP-7 (Fig. 5C). HT-29 human colorectal cancer cells were transfected with vectors encoding the wild-type (SDC2) or mutant versions (D49A, Y51A) of syndecan-2, and then co-immunostained using antibodies against syndecan-2 and MMP-7 followed by Texas Red- and fluorescein isothiocyanate-conjugated secondary antibodies. We found that the levels of syndecan-2 and the membrane localization of MMP-7 were significantly elevated in HT-29 cells expressing SDC2 or D49A. In contrast, the Y51A mutant did not enhance the membrane localization of MMP-7 (Fig. 5C), suggesting that Tyr51 regulates the interaction of syndecan-2 with pro-MMP-7 at the cell surface. Taken together, these data suggest that syndecan-2 directly interacts with the pro-domain of MMP-7, and that this interaction is mediated by Tyr51.

### The Tyr51-mediated syndecan-2 interaction mediates the processing of pro-MMP-7 into active MMP-7

**Figure 6:**
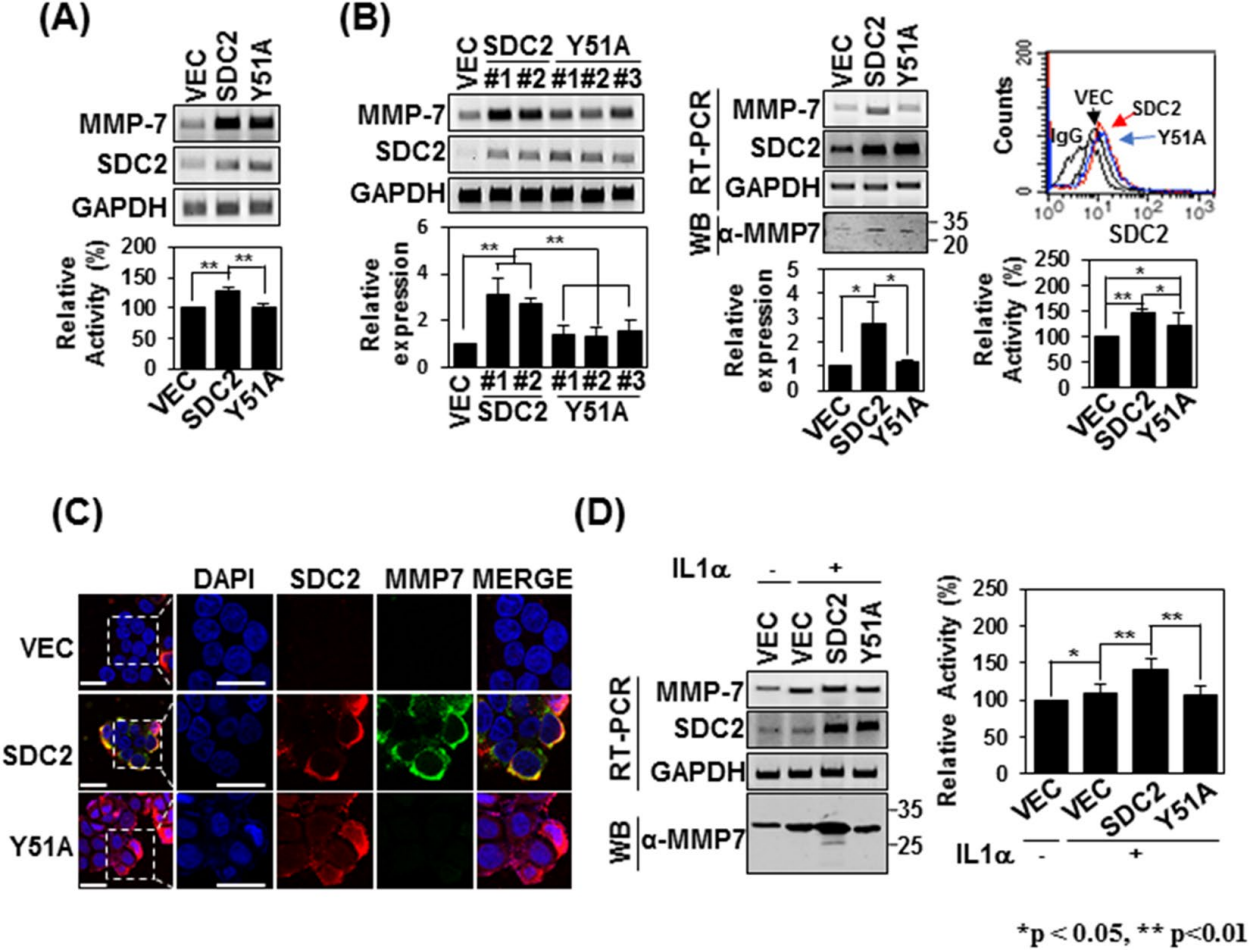
Tyrosine 51 of the syndecan-2 extracellular domain is involved in regulating pro-MMP-7 activation. (**A**) HT-29 cells were transiently transfected with 1 µg of vectors encoding SDC2 or the interaction-defective syndecan-2 mutant, Y51A, and the mRNA expression levels of SDC2 and MMP-7 were evaluated by RT-PCR (top). Conditioned media (CM) were collected and proteolytic activity was measured using quenched fluorescence peptide cleavage assay. The relative activity was normalized versus the fluorescence of a vector control (bottom). Data are shown as mean ± S.D. (n = 3), **p < 0.01 versus VEC or SDC2. (**B**) HT-29 cells were stably transfected with vectors encoding SDC2 or Y51A. The expression levels of the target mRNAs were analyzed by RT-PCR and quantitative real-time PCR (q-PCR) of three independent experiments was performed and normalized to GAPDH expression. Data are shown as mean ± S.D. (n = 3), *p < 0.05, **p < 0.01 versus VEC or SDC2 (left). Flow-cytometric analysis was used to examine membrane-bound SDC2 and Y51A (right top). CM were collected and proteolytic activity was measured using quenched fluorescence peptide cleavage assay (right bottom). (**C**) Control HT-29 cells (VEC) and HT-29 cells stably expressing SDC2 or Y51A were immunostained with anti-syndecan-2 or anti-MMP-7. The results were visualized with Texas Red-conjugated goat anti-rabbit (red) or FITC-conjugated goat anti-mouse (green). DAPI was used to stain nuclei (blue). Scale bar, 20 μm. (**D**) The indicated cells were treated with 1 ng/ml of interleukin-1α (IL-1α). The mRNA expression levels of SDC2 and MMP-7 were evaluated with RT-PCR (left top). CM were collected from the indicated cells and immunoblotted with an anti-MMP-7 antibody (left bottom) or subjected to quenched fluorescence peptide cleavage activity assay (right). Data are shown as mean ± S.D. (n = 3), *p < 0.05, **p < 0.01 versus VEC or SDC2.

Since syndecan-2 is known to mediate the activation of pro-MMP-7, we investigated how interrupting their interaction could affect syndecan-2-mediated MMP-7 activation. Firstly, we investigated whether the expression of syndecan-2 affects that of MMP-7. As shown in Fig. 6A, transient expression of Y51A mutant syndecan-2 increased MMP-7 mRNA expression to levels comparable to those seen with syndecan-2. Interestingly, when we analyzed the enzymatic activity in conditioned media (CM) obtained from HT-29 cell cultures, we found that this activity was increased by overexpression of syndecan-2 but this increase was blocked in cells expressing the Y51A mutant (Fig. 6A). This suggests that Tyr51 is crucial for the syndecan-2 interaction and the activation of pro-MMP-7. To further analyze the involvement of Tyr51 in the activation of pro-MMP-7, we generated HT-29 cells stably overexpressing syndecan-2 or the Y51A mutant (Fig. 6B). Interestingly, all stable clones expressing the Y51A mutant showed slightly less protein expression of MMP-7 compared to cells expressing syndecan-2, although there was no between-group difference in the mRNA expression or cell-surface localization of MMP-7. Similar to syndecan-2-expressing cells, those overexpressing the Y51A mutant still showed lower enzymatic activity of MMP-7 in the conditioned medium (Fig. 6B) and much reduced membrane localization of MMP-7 (Fig. 6C), compared to control cells. Cells of both groups exhibited upregulation of MMP-7 when treated with IL-1α, which is known to induce MMP-7 expression in colon cancer cells^34^; however, the conditioned medium from IL-1α-treated HT-29 cells stably expressing the Y51A mutant showed significantly less enzymatic activity of MMP-7 (Fig. 6D). These data strongly suggest that Tyr51 in the syndecan-2 extracellular domain mediates both the interaction with and the processing of pro-MMP-7.

### The syndecan-2-pro-MMP-7 interaction mediated by Tyr51 functionally regulates syndecan-2

**Figure 7:**
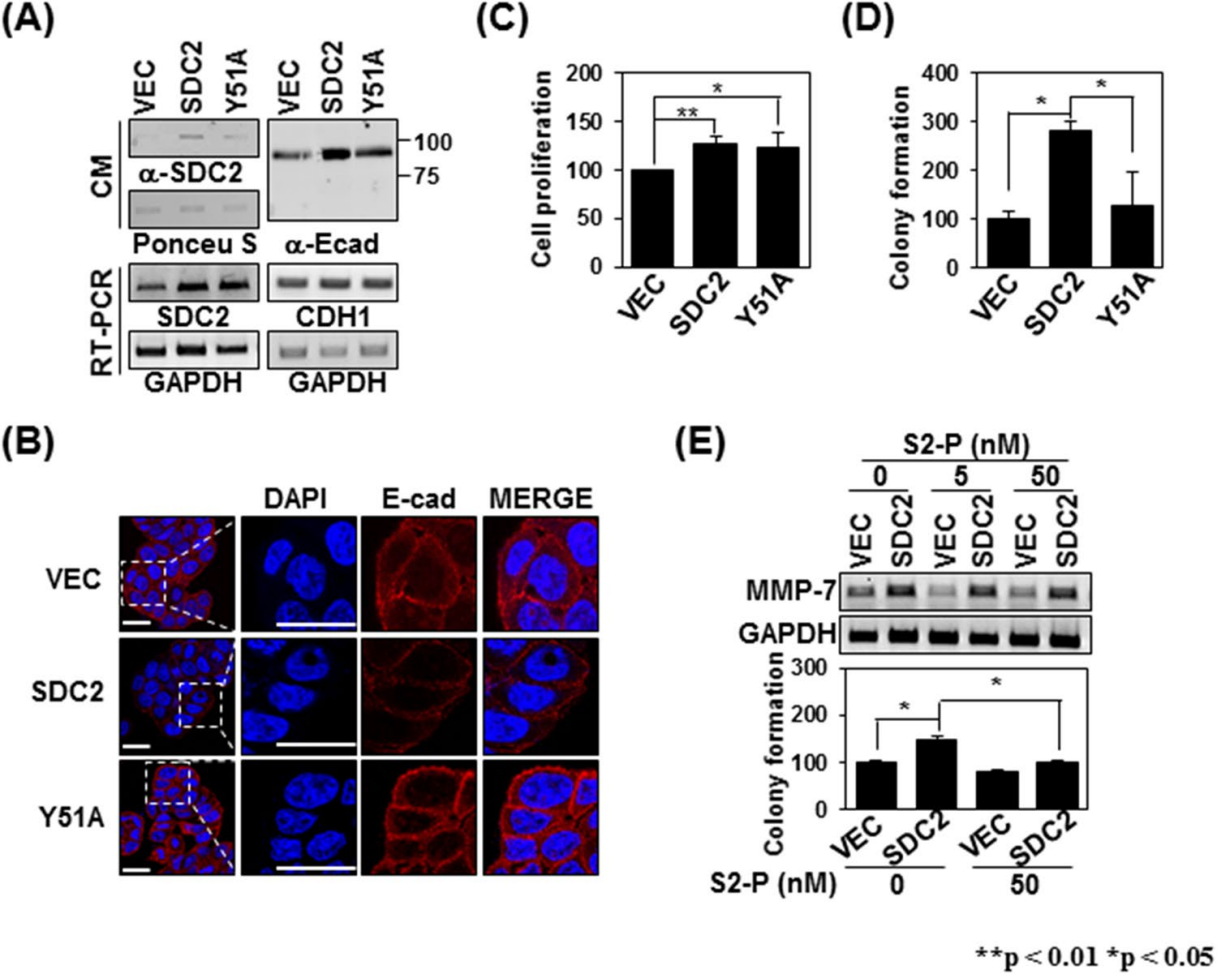
The Tyrosine 51-mediated interaction regulates syndecan-2-mediated tumorigenic activities. (**A**) CM were collected from the indicated cells, immunoblotted with anti-syndecan-2 (left) or anti-E-cadherin (right) antibodies, and subjected to RT-PCR. (**B**) Cells were immunostained with anti-E-cadherin antibody and the results were visualized with Texas Red-conjugated goat anti-rabbit. DAPI was used to stain nuclei (blue). Scale bar, 20 μm. (**C**) The number of cells were evaluated with MTT assay as described in ‘Materials and Methods’. Data are shown as mean ± S.D. (n = 3), *p < 0.05, **p < 0.01 versus VEC or SDC2. (**D**) The indicated cells (1 × 10^5^ cells/well) were seeded on soft agar. After 17 days, colonies were stained with 0.005% crystal violet and counted. Data are shown as mean ± S.D. (n = 3); *p < 0.05 versus VEC or SDC2. (**E**) HT-29 cells stably expressing syndecan-2 were treated with 0, 5, and 50 nM S2-P peptide. At 24 h post-treatment the expression levels of the target mRNAs were analyzed by RT-PCR. GAPDH was used as a control (top). Cells (1 × 10^5^ cells/well) were seeded in soft agar with or without 50 nM S2-P peptide, allowed to grow for 17 days, and the number of viable colonies was counted. Data are shown as mean ± S.D. (n = 3), *p < 0.05 versus VEC or un-treatment S2-P peptide.

It is well known that MMP-7 processes several cell surface receptors^35,36^ and we previously reported that syndecan-2-mediated MMP-7 activation regulates the extracellular shedding of syndecan-2^37^ and E-cadherin^36^. Therefore, we investigated whether expression of the Y51A mutant defective in the syndecan-2-MMP-7 interaction led to alterations in the extracellular domain shedding of syndecan-2 and/or E-cadherin. When we analyzed the shed fragments of syndecan-2 and E-cadherin in conditioned medium, we found that overexpression of syndecan-2 increased the levels of shed syndecan-2 and E-cadherin in the conditioned medium, but overexpression of the Y51A mutant has much less ability to increase these levels (Fig. 7A). Immunohistochemical staining with anti-E-cadherin extracellular-domain antibody showed that the total cell surface expression of E-cadherin was much lower in Y51A-overexpressing HT-29 cells compared to those overexpressing syndecan-2 (Fig. 7B), supporting the idea that the activity of MMP-7 is reduced in Y51A-overexpressing cells. Since syndecan-2 plays an important role in regulating the tumorigenic activities of colon cancer cells^16,28,29,37^, we next investigated whether the reduced activation ability of the Y51A mutant affected the tumorigenic activity of colon cancer cells. Whereas no significant difference in cell proliferation of Y51A-overexpressing HT-29 cells compared to those overexpressing syndecan-2 (Fig. 7C), overexpression of the Y51A mutant reduced syndecan-2-mediated colony formation (Fig. 7D), which are dependent on MMP-7 activity. Consistent with these findings, the addition of S2-P, which inhibited the interaction of syndecan-2 with the pro-domain of MMP-7, reduced the syndecan-2-mediated colony forming activity of HT-29 cells (Fig. 7E). Taken together, our data strongly suggest that Tyr51 in the extracellular domain of syndecan-2 specifically mediates its interaction with and activation of pro-MMP-7.

## Discussion

Although we previously showed that the extracellular domain of syndecan-2 interacts with the pro-domain of MMP-7^16^, the structural basis of this interaction was unknown. As shown in Fig. 1, we herein first confirmed that human syndecan-2 extracellular domain interacted with the pro-domain of MMP-7 in our experimental system and then used serial deletion mutants of the syndecan-2 extracellular domain to reveal that its N-terminal region (amino acid residues 41 to 60, recapitulated in synthetic peptide S2-P) directly interacted with the pro-domain of MMP-7 (Fig. 1B). S2-P competitively inhibited the interaction between recombinant syndecan-2 extracellular domain and the pro-domain of MMP-7 (Fig. 1C), confirming that this sequence is involved in the interaction with MMP-7. With respect to the pro-domain of MMP-7, which is composed of three helices with flexible linker regions, our NMR data showed that S2-P peptide binding triggered chemical shift perturbations in parts of the α1, α2, and α2–3 helices, suggesting that the overall structural folding of the pro-domain is important for the interaction of pro-MMP-7 with syndecan-2 (Fig. 3B). Our biochemical data obtained using recombinant proteins revealed that the α2–3 helix, but not the α1, α2, or α1–2 helixes, interacted with recombinant syndecan-2 *in vitro* (Figs 2, 4), suggesting that the α2–3 helix in the pro-domain of MMP-7 plays an important role in the interaction with the syndecan-2 extracellular domain. We used the HEX 6.3 and HADDOCK modeling programs to calculate a docking model of the interaction between the pro-domain of MMP-7 and S2-P; our results suggested that a cluster of hydrophobic surface residues forms a ligand-binding site for the side chain of syndecan-2 Tyr51 to interact with a hydrophobic alanine from the pro-domain of MMP-7, and that this creates a highly favorable hydrophobic interaction between protein and ligand molecules (Fig. 5). The base of the pocket, which is located in the flexible linker region that joins helixes α2 and α3, is lined with purely hydrophobic residues with the exception of Glu32 (Figs 3, 5). Our modeling results thus suggest that conserved Tyr51 of syndecan-2 is important for its interaction with the pro-domain of MMP-7 (Fig. 5). Indeed, replacement of Tyr51 with alanine (Y51A) significantly reduced the interaction between syndecan-2 and the pro-domain of MMP-7 (Fig. 5A,B), and the Y51A mutant failed to colocalize with MMP-7 at the cell surface (Fig. 5C). In addition, our docking model indicates that syndecan-2 forms electrostatic interactions with the pro-domain of MMP-7, which is consistent with the results of our NMR titration and fluorescence analyses. In the future, it may be useful to generate and analyze mutations of other interacting charged residues to further delineate their molecular roles.

Since syndecan-2 is known to mediate the activation of pro-MMP-7, we further investigated the effect of the identified interaction on syndecan-2-mediated MMP-7 activation (Fig. 6). As expected, transient overexpression of the Y51A mutant yielded less enzymatic activity in the conditioned media of HT-29 cells, compared with that obtained from cells expressing a comparable level of wild-type (WT) syndecan-2 (Fig. 6A). This supports the notion that the Tyr51-mediated interaction is crucial for both the interaction of syndecan-2 with pro-MMP-7 and its ability to activate the enzyme. A similar effect was detected in HT-29 cells stably expressing the Y51A mutant (Fig. 6B), and both mutant- and WT-overexpressing cells showed reductions of MMP-7 enzymatic activity in response to IL-1α treatment (Fig. 6D). This reduced enzymatic activity was associated with reduced extracellular domain shedding of syndecan-2 and E-cadherin (Fig. 7A), which are well-known substrates for MMP-7 in cancer cells^36^. These data strongly suggest that Tyr51 in the syndecan-2 extracellular domain mediates both its interaction with and the processing of pro-MMP-7.

Interestingly, all stable Y51A-expressing clones expressed slightly less MMP-7 compared to cells stably expressing WT syndecan-2 (Fig. 6B). This suggests that syndecan-2 and the Y51A mutant induced MMP-7 expression at a similar rate at an early stage of syndecan-2 transfection, but the sustained levels of MMP-7 expression came to differ over time. We previously reported that MMP-7 cleaved the extracellular domain of syndecan-2, and that shed syndecan-2 can induce the expression of MMP-7 as a cell-surface ligand^37^. Therefore, during stable expression, the degree of MMP-7 expression is influenced by both syndecan-2 and shed syndecan-2, and these actions are mediated by active MMP-7. We believe this explains the difference seen in the MMP-7 expression levels of transiently and stably transfected cells. A similar phenomenon was previously observed with a non-cleavable syndecan-2 mutant (NC), which has reduced ability to generate shed syndecan-2 by MMP-7^37^.

Since the interaction of MMPs with their cell-surface receptors is known to be important in regulating cancer activity^16,25,26^, we further investigated whether the Tyr51-mediated interaction described herein contributes to regulating cancer activity (Fig. 7). Indeed, expression of the interaction-defective syndecan-2 mutant (Y51A) decreased the syndecan-2-mediated anchorage-independent growth of HT-29 cells (Fig. 7D). Meanwhile, addition of the synthetic peptide (S2-P) reduced the syndecan-2-mediated anchorage-independent growth of HT-29 cells in parallel with its ability to reduce the interaction of syndecan-2 with pro-MMP-7 (Fig. 7E), suggesting that the ability of syndecan-2 to regulate cancer activity regulation is closely related to its interaction with pro-MMP-7. Since it is known that MMP-7 is localized at invasive front in colon cancer cells, syndecan-2 could immobilize pro-MMP-7 to restrict its range of action and regulate the processing of pro-MMP-7 to further regulate degradation of ECM and cell adhesion molecules. This finding is consistent with our previous finding that syndecan-2 induces extracellular release of E-cadherin and supports the acquisition of a fibroblast-like morphology by regulation of MMP-7 in a colon cancer cell line^38^.

In summary, we herein reveal the structural basis of the interaction between the syndecan-2 extracellular domain and the pro-domain of MMP-7. Our data show that Tyr51 in the extracellular domain of syndecan-2 (residues 41 to 60) mediates the interaction of syndecan-2 with the α2 helix-loop-α3 helix in the pro-domain of MMP-7, and that this interaction is critical for the localization of pro-MMP-7 on the cell surface, its processing into active MMP-7, and the syndecan-2-mediated regulation of tumorigenic activity of colon cancer cells. Although future studies will be required to fully elucidate the mechanisms through which syndecan-2-induced MMP-7-mediated signaling affects colon cancer cells, our present findings provide important new insights into the regulation of colon cancer mediated by syndecan-2 as a docking receptor at the cancer cell surface.

## Materials and Methods

### Antibodies and materials

A monoclonal antibody capable of recognizing human, rat, and mouse syndecan-2 was produced by AdipoGen Inc. (Incheon, Korea) using the Fc-fused extracellular domain of syndecan-2^16^. A polyclonal antibody that recognize human, rat, and mouse syndecan-2 was produced by AbClon (Seoul, Korea) using a human syndecan-2 extracellular domain peptide. Polyclonal anti-E-cadherin and anti-His tag antibodies, and monoclonal anti-MMP-7 and anti-GST antibody were purchased from Santa Cruz Biotechnology (Santa Cruz, CA, USA). The 2,2,2-trifluoroethanol (TFE) was purchased from Sigma-Aldrich (St Louis, MO, USA). IL-1α was purchased from R&D Systems (Minneapolis, MN, USA).

### Peptide synthesis

The peptide corresponding to human syndecan-2 extracellular domain residues 41–60 (S2-P: SGVYPIDDDDYASASGSGAD) was synthesized using an improved version of the Fmoc chemistry-based solid-phase method (Anygen Inc., Kwangju, Korea)

### Cell culture and transfection

The HT-29 human colon adenocarcinoma cell line (ATCC®HTB-38^TM^) was purchased from ATCC (Manassas, VA, USA) and maintained in McCoy’s 5 A complete medium (Welgene, Daegu, Korea) supplemented with 10% (v/v) fetal bovine serum (FBS; Hyclone, Logan, UT, USA) and gentamycin (50 g/ml; Sigma-Aldrich, St Louis, MO, USA) at 37 °C in a 5% CO_2_-containing humidified atmosphere. Transfections were performed using the Viva Magic transfection reagent (Vivagen, Gyeonggi-Do, Korea) according to the manufacturer’s instructions. HT-29 cells (4.0 × 10^5^ cells/well) were plated to 6-well plates, incubated at 37 °C for 24 h, and then transfected with the various expression vectors. To generate cell lines stably expressing the various versions of syndecan-2, HT-29 cells (1 × 10^6^) were transfected with 1 μg of the indicated expression vectors and then selected for 4 weeks in medium containing 800 μg/ml G418 (EMD Biosciences, San Diego, CA, USA). The surviving clones were individually isolated and analyzed by fluorescence-activated cell sorting (FACS) and reverse transcription polymerase chain reaction (RT-PCR).

### Vector construction

A series of extracellular domain deletion mutants of syndecan-2 (S2E, S2E-N, S2E-NI, S2E-NII, and S2E-C) were constructed by PCR amplification and cloned into the pGEX-5 × −1 vector (Amersham Biosciences, Piscataway, NJ, USA). The pro-domain of MMP-7 and its deletion mutants (ΔN, ΔC, ΔNC, α1, α2, α1–2, and α2–3) cloned into the pET32a(+) vector (Novagen, Madison, WI, USA).

### Site-directed mutagenesis

Site-directed mutagenesis of syndecan-2 (nucleotide: ^139^GATGAC**GAT**GAC**TAC**GCTTCT^159^, amino acid: ^47^DD**D**D**Y**AS^53^) in the pcDNA3.0 vector was performed using a Transformer site-directed mutagenesis kit (Stratagene, La Jolla, CA, USA). The synthetic oligonucleotide, ^139^GATGAC**GCT**GACTACGCTTCT^159^ (Asp49 → Ala; ^47^DD**A**DYAS^53^), was used to change the indicated amino acids. To mutate Tyr51 to alanine (nucleotide: ^139^GATGACGATGAC**GCC**GCTTCT^159^, amino acid: ^47^DDDD**A**AS^53),^ point mutants were introduced into the extracellular domain of syndecan-2 by commercial gene synthesis (Bioneer, Daejeon, Korea).

### Expression and purification of recombinant proteins

To express GST-fused syndecan-2 proteins were purified with glutathione-agarose beads (GE Healthcare Life Sciences, Piscataway, NJ, USA), as described previously^39^. To express His-tagged proteins, we used pET32a + encoding MMP-7 fusion proteins were purified with Ni-NTA agarose columns (Qiagen, Hilden, Germany). Briefly, cell pellets were harvested, lysed with lysis buffer (20 mM sodium phosphate, 500 mM NaCl, and 5 mM β-mercaptoethanol, pH 6.0) and applied to Ni-NTA affinity columns. Each column was washed twice (20 mM sodium phosphate, 500 mM NaCl, 5 mM β-mercaptoethanol, pH 6.0, and 30 mM imidazole) and the target proteins were eluted (20 mM sodium phosphate, 500 mM NaCl, 5 mM β-mercaptoethanol, pH 6.0, and 500 mM imidazole).

### Structural analysis by circular dichroism spectroscopy

Circular dichroism (CD) spectra were recorded on a JASCO J-810 spectropolarimeter (Jasco, Tokyo, Japan) calibrated with ammonium D-10-camphorsulfonate at 290 nm and equipped with a thermostatically controlled cell holder attached to a water bath with an accuracy of ± 0.1 °C. The parameters utilized for far-ultraviolet (UV) CD measurements were as follows: cell path length of 0.1 cm for scanning between 250 − 190 nm; collection at a bandwidth of 1 nm; scan speed of 50 nm/min^−1^, signal-averaged over at least eight scans; and baseline correction performed by subtracting the spectrum of the buffer. CD spectral analyses CD spectra were processed using CD tool software^40^. Secondary structure analyses were performed with the Web Address for CDPro Web server^40^ using the following algorithms: quantitative estimations of the secondary-structure content were made with the CDPro software package, CONTINLL, SELCON3 and CDSSTR^40–42^. A goodness-of-fit parameter (Normalized Root Mean Square Deviation; NRMSD)^43^ was calculated for all methods that produce back-calculated spectra (CONTINLL, SELCON3, CDSSTR).

### Fluorescence assay

The binding affinity between the pro-domain of MMP-7 (His-PD) and S2-P peptide was measured at 298 K (25 °C) using an LS55 fluorescence spectrophotometer (Perkin Elmer, Waltham, MA) with 280 nm excitation and 260–450 nm emission values. Both His-PD and S2-P peptide were prepared in PBS buffer at pH 7.4. The concentration of His-PD was 15 uM and S2-P peptide was titrated to His-PD up to 1:200 molar ratios using a thermostat cuvette. The dissociation constant (K_*d*_) of the His-PD /S2-P peptide complex was calculated using the equation, log[(Fo-F)/F] = log(1/Kd) + nlog[ligand], where Fo and F represent the fluorescence intensity of His-PD at 347 nm in the absence and presence of S2-P peptide, respectively. n represent the number of binding sites.

### NMR experiment and titration

The human pro-MMP-7 N/C-terminal truncated mutant NMR samples were prepared in 20 mM Na_2_HPO_4_, pH 6.0, 5 mM β-mercaptoethanol, 1 mM sodium azide, 100 mm dodecylphosphocholine, and 10% D_2_O. NMR spectra were obtained at 283 K on Bruker AVANCED RX 850 MHz and 900 MHz spectrometers using a Cry probe (Division of Magnetic Resonance, Korea Basic Science Institute (KBSI), Ohchang, Chungbuk; National Center for Inter-university Research Facilities, Seoul National University, Korea). The data were processed and analyzed using the NMRPipe/NMRDraw (Biosym/Molecular Simulation, Inc., San Diego, CA) and Sparky programs (University of California, San Francisco, CA). Sequential backbone resonance assignment was performed on data obtained from ^1^H-^15^N heteronuclear single quantum coherence (HSQC), three-dimensional HNCACB, and CBCA(CO)NH experiments. NMR titrations were performed using ^15^N-labeled pro-domain of MMP-7 truncation mutant and the S2-P peptide at different molar ratios (1:15). Chemical shift change values were calculated using the equation Δδ_AV_ = ((Δδ_1H_)^2^ + (Δδ_15N_/5)^2^)^½^, where Δδ_AV_, Δδ_1H_, and Δδ_15N_ represent the average chemical shift value, proton chemical shifts, and nitrogen chemical shift changes, respectively.

### Complex docking model

An atomic-resolution structure of proMMP-7 was constructed using the solution structures of human MMP-7 (PDB: 2MZE) and the homology modeling method applied by Modeller 9^44^. The conformation of the S2-P peptide was predicted using PEP-FOLD3^45^. The constructed proMMP-7 and S2-P structures were used as the receptor and ligand, respectively, for docking by HEX 6.3^31^. The parameters for docking were as follows: correlation type, shape only or shape plus electrostatics; post processing, MM Minimization; grid dimension, 0.6 Å; receptor range, 180°; ligand range, 180°; receptor and ligand step size, 7.5°; twist range, 360°; and twist step size, 5.5°; distance range, 40 Å; scan step, 0.8 Å. The docking model generated for proMM7 and S2-P was then analyzed using Chimera^46^ and VMD^47^, and a refined docking model was obtained using the HADDOCK program^32^ based on the results of our NMR titration experiments. In the receptor molecule, Glu32, Gly48, and Ser52 were assigned as residues that actively participate in the interaction with SDC2. The passive residues were assigned automatically around the active residues. The active and passive residues of the ligand molecule, SDC2, were assigned based on electrostatic potential. From 200 clusters of the water-refined HADDOCK models, seven significant clusters were generated with various statistics. The root mean square deviation (RMSD) from the overall lowest energy structure, was 0.9 ± 0.6 Å.

### MMP enzyme activity assay

The catalytic activity of MMP-7 was analyzed by a peptide cleavage assay using the quenched fluorescent peptide, (7-methoxycoumarin-4-yl) acetyl-Pro-Leu-Gly-LeuN-3(2,4-dinitrophenyl)-L-2,3 diaminopropionylAla-Arg-NH2 (Bachem, Bubendorf, Switzerland), as a substrate. The reactions were performed in a final volume of 100 μl in MMP assay buffer (20 mM Tris HCl, pH 7.4, 150 mM NaCl, 5 mM CaCl_2_, 0.5 mM ZnCl_2_, 0.001% Brij35) in the presence of 1 μM oligopeptide and 50 μl of CM for 4 h at 37 °C. The reaction was stopped by the addition of 0.1 M (final concentration) sodium acetate, pH 4.0. Fluorescence was determined at an excitation wavelength of 328 nm and an emission wavelength of 393 nm using a SpectraMax® i3 plate reader (Molecular Devices, Sunnyvale, CA, USA)^48^.

### Immunofluorescence analysis

Cells cultured on coverslips in 12-well plates (2.0 × 10^5^ cells/well) were fixed with 3.5% paraformaldehyde for 10 min, washed with PBS, blocked with 0.5% BSA, and incubated overnight with specific antibodies at 4 °C. The cells were then washed with PBS and incubated FITC-conjugated secondary antibodies (Thermo Fisher Scientific, Waltham, MA, USA) for 1 h at 25 °C. The coverslips were mounted on glass slides with mounting solution containing 4′,6-diamidino-2-phenylindole (DAPI), and the results were imaged under a confocal fluorescence microscope (Carl Zeiss, Gottingen, Germany)^48^.

### Flow cytometry

HT-29 cells (90% cell confluency) were washed with PBS, released by the addition of 5% FBS and 1 mM EDTA in PBS, collected by centrifugation, resuspended in PBS, and incubated with anti-syndecan-2 in PBS containing 10% FBS for 1 h at 4 °C. The cells were then washed three times with PBS containing 0.05% Tween-20, and incubated with either FITC-conjugated anti-mouse IgG (Thermo Fisher Scientific, Waltham, MA, USA), in PBS containing 10% FBS for 1 h at 25 °C. The expression of syndecan-2 was analyzed by flow cytometry counts of 1 × 10^4^ cells (FACS Calibur; BD Bioscience, San Diego, CA, USA)^48^.

### Colony forming assays

Each well of a 6-well culture plate was coated with 3 ml of bottom agar mixture (McCoy’s 5 A, 10% FBS, 0.6% agar). After the bottom layer had solidified, 1 ml of top agar mixture (McCoy’s 5 A, 10% FBS, 0.3% agar) containing HT-29 cells (1 × 10^5^ cells/well) was added to each well and the cultures were incubated at 37 °C in a 5% CO_2_ atmosphere. Colony formation was monitored daily with a light microscope. After 17 days, the colonies were stained with 0.005% crystal violet and photographed with a digital camera^48^.

### TCA precipitation and slot blotting

CM were collected and mixed with 100% TCA (Yakuri Pure Chemicals, Osaka, Japan) at a 1:10 ratio. Each mixture was incubated for 30 min at 4 °C, pelleted, washed with 10% TCA, dried, and then dissolved in 0.1 N NaOH^48^.

### RT-PCR and Quantitative real-time PCR

Total RNA was isolated from cells using easy-BLUE kit (Intron, Seoul, South Korea). The RNA was extracted with chloroform and precipitated with isopropanol. The RNA pellet was washed with 75% ethanol and resuspend in DEPC-treated water. Approximately 3 μg of RNA was used to generate cDNA using AMV Reverse Transcriptase (Cat# M5108) and Random Primer (Cat# C1181) (Promega US, Madison, WI, USA). Aliquots of the resulting cDNAs were amplified using the primers sequences given in Table 1. After an initial denaturation at 94 °C for 5 min, the samples were subjected to 30 cycles of denaturation at 94 °C for 30 seconds, annealing at 55 °C for 60 seconds, and extension at 72 °C for 60 seconds. Human GAPDH was amplified as an internal control. The generated PCR products were separated by 1% agarose gel electrophoresis. Quantitative real-time PCR was performed using the CFX96™ Real-Time PCR Detection System (Bio-Rad) in a two-step procedure using SensiFAST™ SYBR® Hi-ROX Kit (BioLine, London, UK). GAPDH was amplified as an internal control. Using the primers sequences are given in Supplementary Table 1. All reactions were performed in a 96-well plate using the following cycling conditions: 40 cycles of 95 °C for 15 seconds, 60 °C for 30 seconds, and 72 °C for 1 min. Using the CT (ΔΔCT) method, the value of each control sample was set at 1 and used to calculate the fold-change of the target genes^48^.

**Table 1:** Primer sequences used in the PCR.

PCR	Human Gene	Forward primers (5′–3′)	Reverse primers (5′–3′)
RT-PCR	SDC-2	ACATCTCCCCTTTGCTAACGGC	TAACTCCATCTCCTTCCCCAGG
	MMP-7	GGTCACCTACAGGATCGTATCATAT	CATCACTGCATTAGGATCAGAGGAA
	CDH-1	TCATGAGTGTCCCCCGGTAT	TCTTGAAGCGATTGCCCCAT
	GAPDH	CCACCCATGGCAAATTCCATGGCA	TCTAGACGGCAGGTCAGGTCCACC
qPCR	SDC-2	CTGCCCCTAAACTTCTGCCGT	CTTGTTGGTTTCTGCACTCCC
	MMP-7	GGCTTTAAACATGTGGGGCA	GGCCCATCA AATGGGTAGGA
	GAPDH	CCTCAAGATCATCAGCAAT	CCATCCACAGTCTTCTGGGT

### Cell proliferation assay

Cell proliferation was measured using the MTT [3-(4,5-dimethythiazol-2-yl) 2,5-diphenyltetrazolium bromide; Amresco, Solon, OH, USA] assay. In brief, after HT-29 cells (5,000 cells/well) were incubated for 48 h, medium containing 0.5 mg/ml MTT was added to each well, and the cells were incubated for additional 1 h. The medium was then removed and 100 μl of acidic isopropanol (90% isopropanol, 0.5% sodium dodecyl sulfate (SDS) and 25 mM NaCl) was added to each well. The mean concentration of absorbance at 570 nm in each sample set was measured using a 96-well microtiter plate reader (Dynatech, Chantilly, VA, USA)^16^.

### Statistical analysis

Data are presented as the means from at least three independent experiments. Statistical analysis was performed using an unpaired Student’s *t* -test. A *p*-value less than 0.05 or 0.01 was considered statistically significant.

## Supplementary information

Tyrosine 51 residue of the syndecan-2 extracellular domain is involved in the interaction with and activation of pro-matrix metalloproteinase-7 

## Data Availability

The datasets generated during and/or analyzed during the current study are available from the corresponding author on reasonable request.
