# Supplementary material for: Tyrosine 51 residue of the syndecan-2 extracellular domain is involved in the interaction with and activation of pro-matrix metalloproteinase-7
Source: Sci Rep. 2019 Jul 23;9:10625. doi: 10.1038/s41598-019-47140-5 (PMC6650482; doi:10.1038/s41598-019-47140-5)
Supplement: Supplementary file 1 — Tyrosine 51 residue of the syndecan-2 extracellular domain is involved in the interaction with and activation of pro-matrix metalloproteinase-7 [file 41598_2019_47140_MOESM1_ESM.pdf]

Supplementary information

Tyrosine 51 residue of the syndecan-2 extracellular domain is involved in the interaction with and activation of pro-matrix metalloproteinase-7

Bohee Jang[1*], Ji-Hye Yun[2*], Sojoong Choi[1], Jimin Park[3], Dong Hae Shin[3], Seung-Taek Lee[2], Weontae Lee[2]@, Eok-Soo Oh[1]@

Raw Data Figures

Figure S1

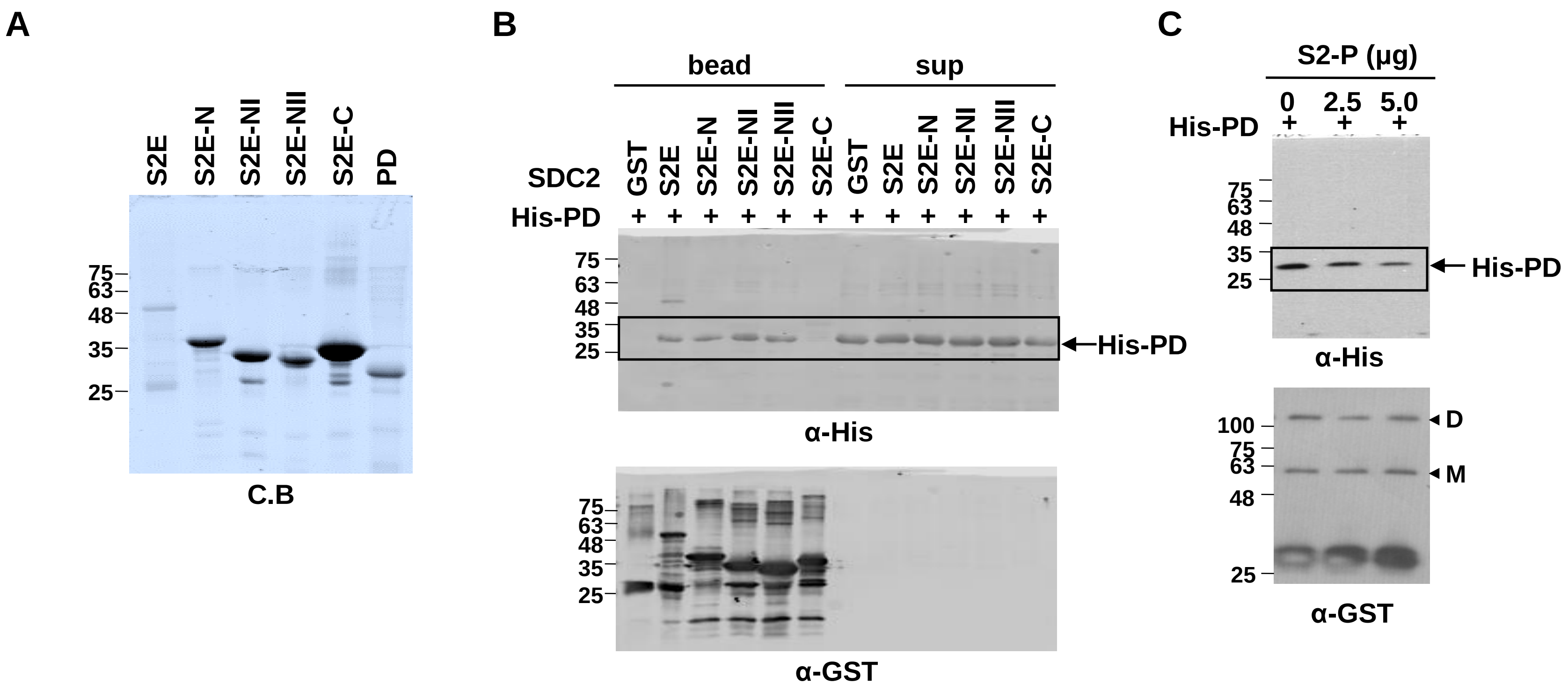

Supplementary Figure 1 : Full-length blots presented in Figure 1.
Raw data of (A) coomassie blue (C.B) stained gel with purified GST-SDC2 mutants and the His-tagged pro-domain of MMP-7. (B,C) Full length western-blots of His- (α-His) and GST-tag (α-GST). (shown as cropped images in Figure 1 A, B and C).

Figure S2

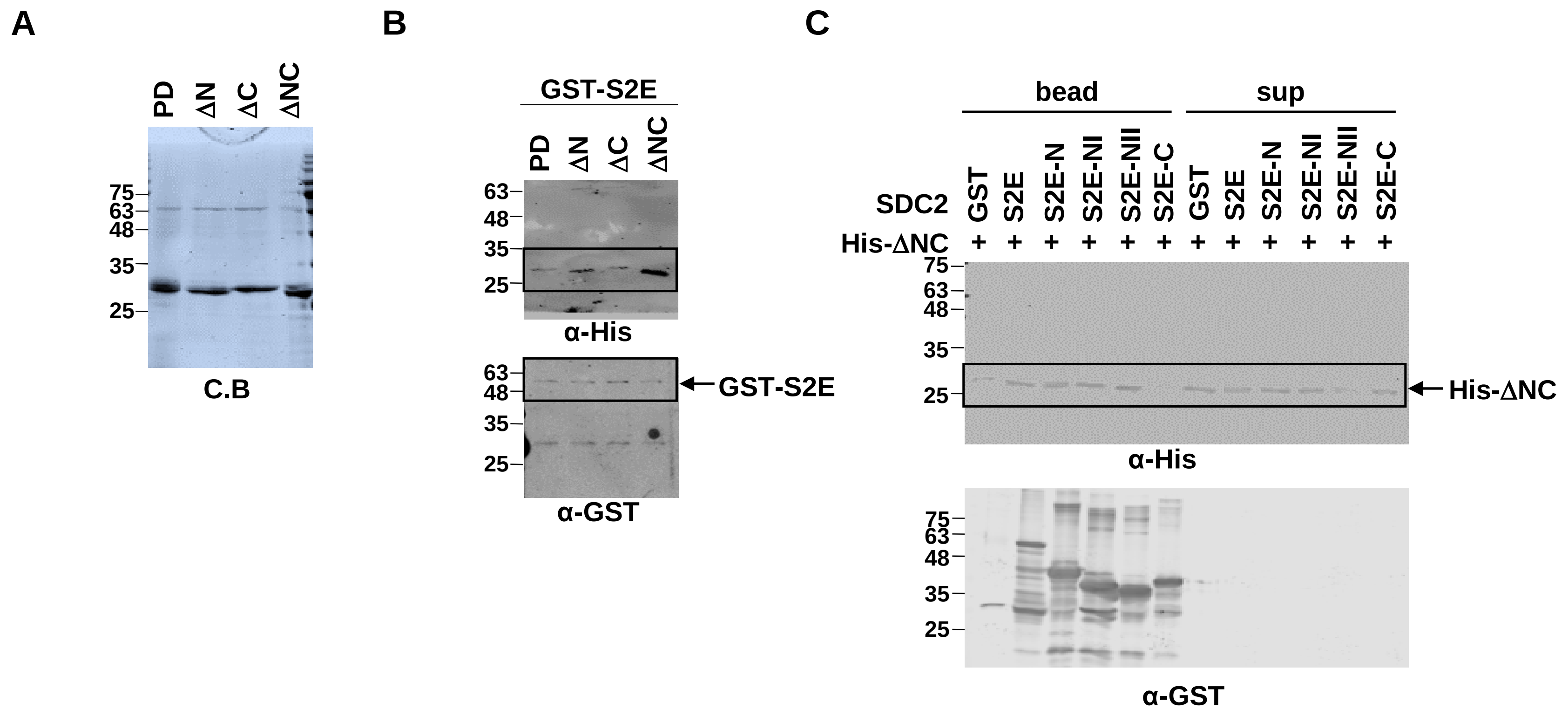

Supplementary Figure 2 : Full-length blots presented in Figure 2.

Raw data of (A) coomassie blue (C.B) stained gel with purified His-tagged pro-domain of MMP-7 mutants. (B,C) Full length western-blots of His- (α-His) and GST-tag (α-GST). (shown as cropped images in Figure 2 A, B and D).

Figure S3

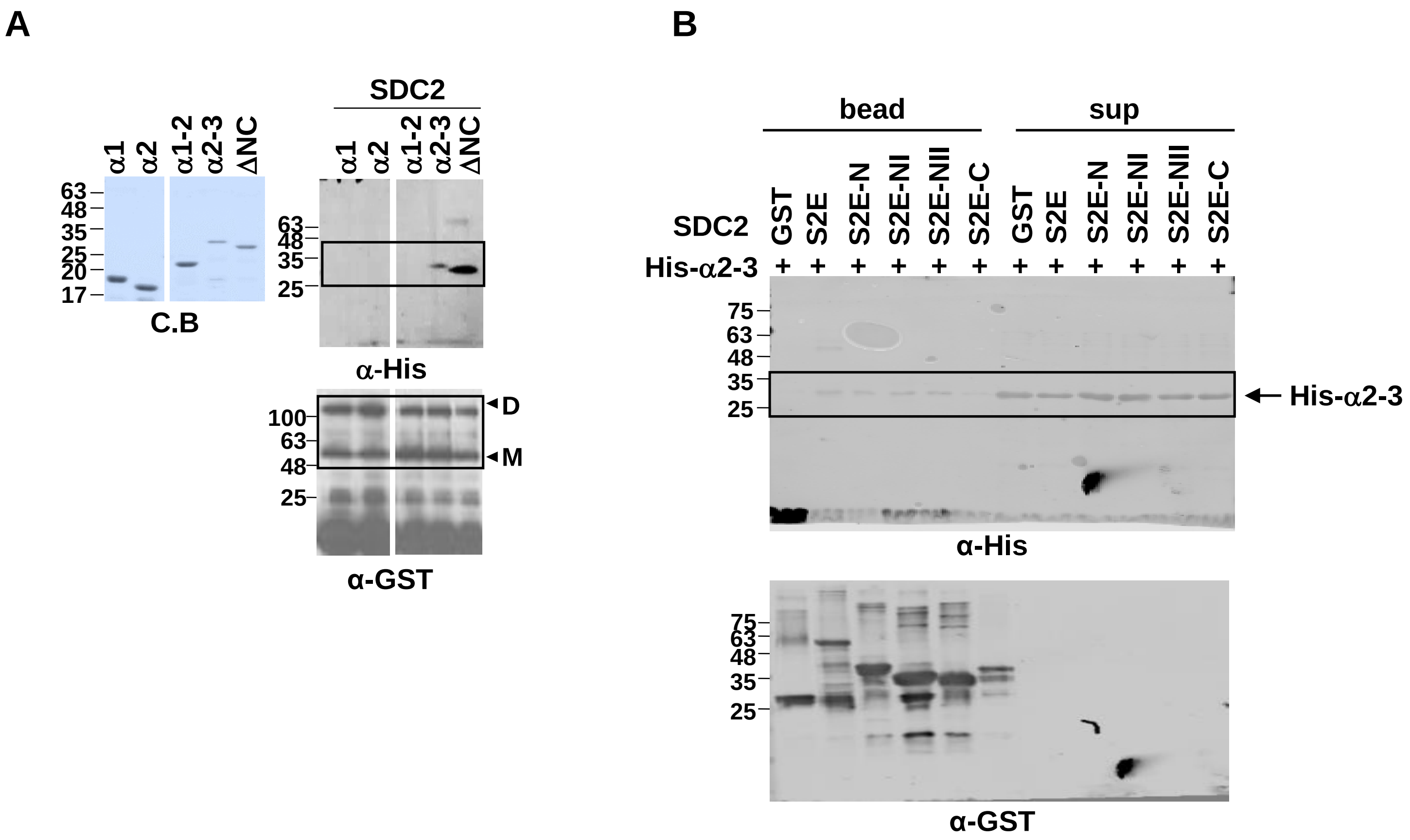

Supplementary Figure 3 : Full-length blots presented in Figure 4.

Raw data of (A) coomassie blue (C.B) stained gel with purified pro-domain of MMP-7 mutants (left panel). Full length of western-blots of His- (α-His) and GST-tag (α-GST, right panel). (B) Full length western-blots of His- (α-His) and GST-tag (α-GST). (shown as cropped images in Figure 4 A, B).

Figure S4

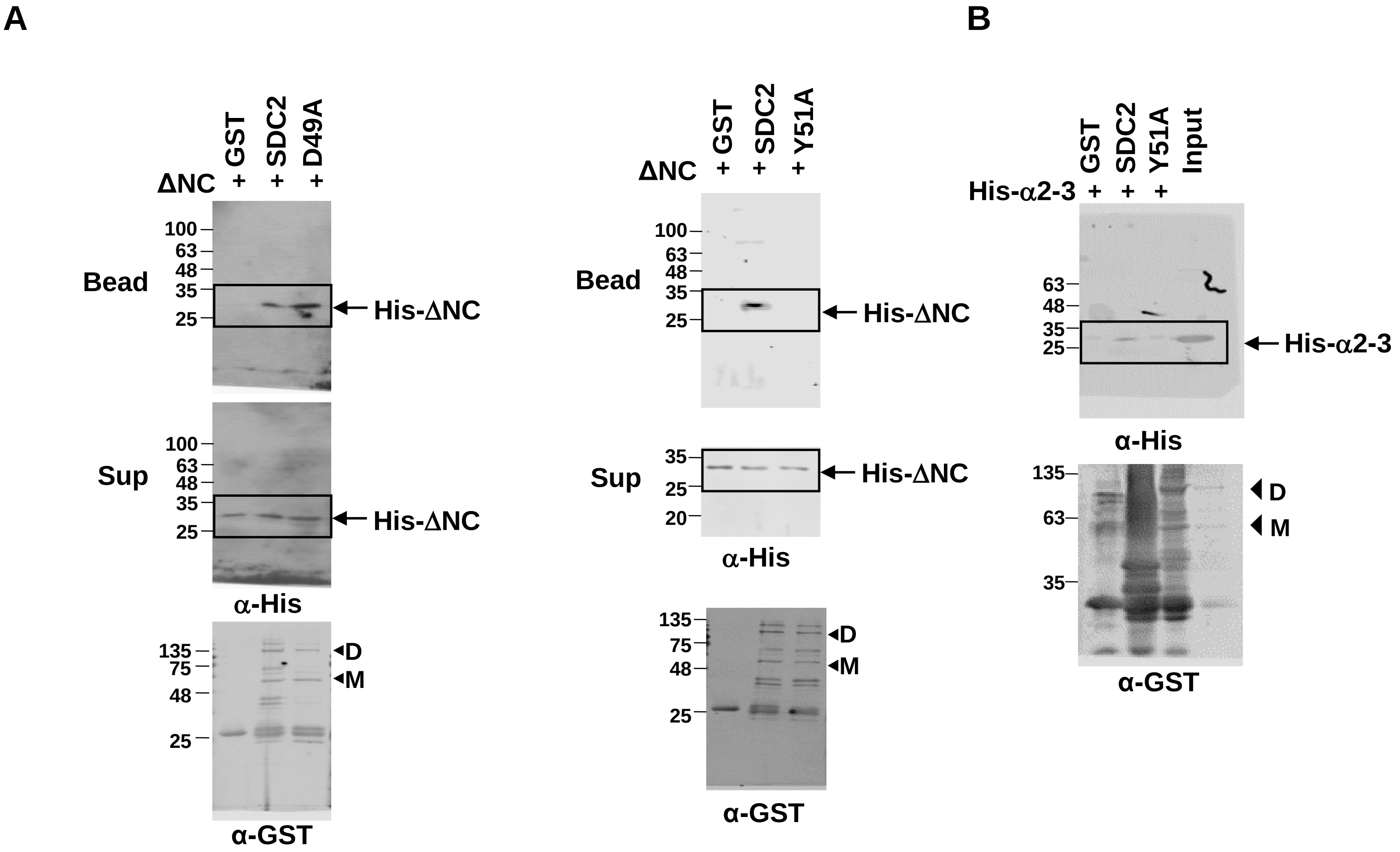

Supplementary Figure 4 : Full-length blots presented in Figure 5.

(A,B) Raw data of full length western-blots of His- (α-His) and GST-tag (α-GST). (shown as cropped images in Figure 5 A, B).

Figure S5

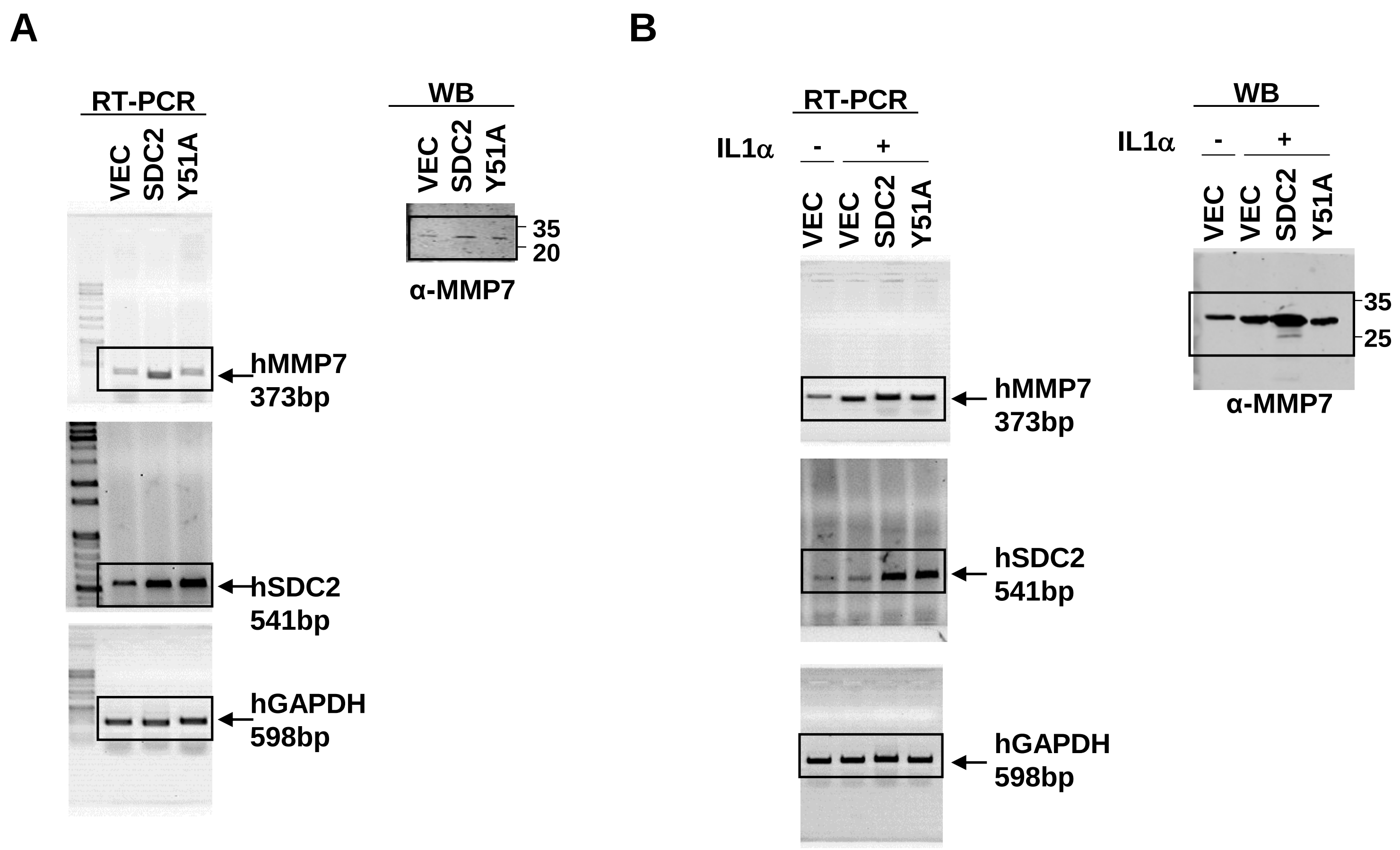

Supplementary Figure 5 : Full-length blots and gels presented in Figure 6.
(A, B) Raw data of full length of agarose gel used for analysis of PCR products (left panel) and full length western-blots of MMP-7 (right panel). (shown as cropped images in Figure 6 B, D).

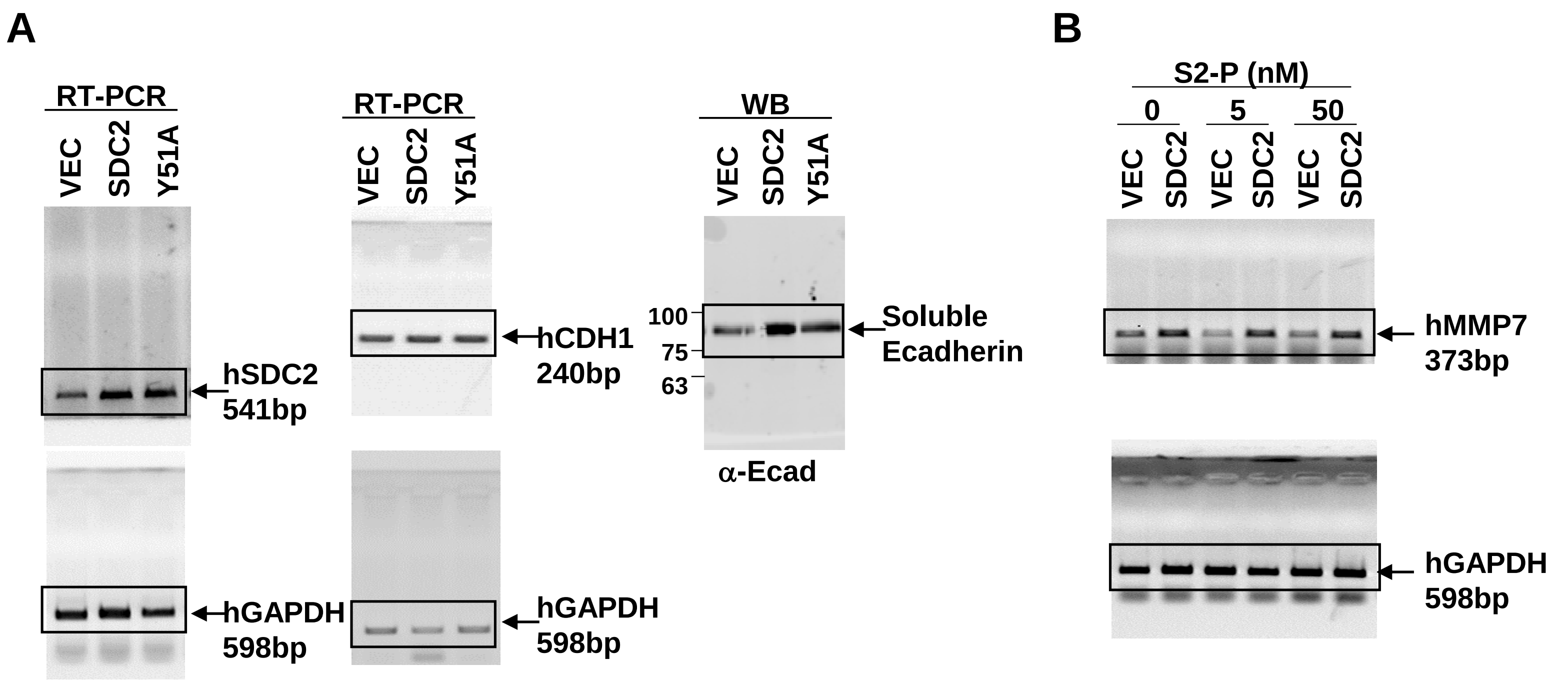

Supplementary Figure 6 : Full-length blot and gels presented in Figure 7.

Raw data of (A) Full length of agarose gel used for analysis of PCR products (left panel) and full length western-blots of E-cadherin (right panel). (B) Full length of agarose gel used for analysis of PCR products. (shown as cropped images in Figure 7 A, E).
